# Discourse on malaria elimination: where do forcibly displaced persons fit in these discussions?

**DOI:** 10.1186/1475-2875-12-121

**Published:** 2013-04-10

**Authors:** Holly A Williams, Heiko Hering, Paul Spiegel

**Affiliations:** 1 International Emergency and Refugee Health Branch, Centers for Disease Control and Prevention, Mail Stop F-60, 4770 Buford Hwy NE, Atlanta, GA, 30341, USA; 2 United Nations High Commissioner for Refugees, Case Postale 2500, CH-1211 Genève 2 Dépôt, Suisse, Switzerland

**Keywords:** Malaria, Malaria elimination, Refugees

## Abstract

**Background:**

Individuals forcibly displaced are some of the poorest people in the world, living in areas where infrastructure and services are at a bare minimum. Out of a total of 10,549,686 refugees protected and assisted by the United Nations High Commissioner for Refugees globally, 6,917,496 (65.6%) live in areas where malaria is transmitted. Historically, national malaria control programmes have excluded displaced populations**.**

**Results:**

The current discourse on malaria elimination rarely includes discussion of forcibly displaced persons who reside within malaria-eliminating countries. Of the 100 malaria-endemic countries, 64 are controlling malaria and 36 are in some stage of elimination. Of these, 30 malaria-controlling countries and 13 countries in some phase of elimination host displaced populations of ≥50,000, even though 13 of the 36 (36.1%) malaria-elimination countries host displaced populations of ≥50,000 people.

**Discussion:**

Now is the time for the malaria community to incorporate forcibly displaced populations residing within malarious areas into malaria control activities. Beneficiaries, whether they are internally displaced persons or refugees, should be viewed as partners in the delivery of malaria interventions and not simply as recipients.

**Conclusion:**

Until equitable and sustainable malaria control includes everyone residing in an endemic area, the goal of malaria elimination will not be met.

## Introduction

The plight of forcibly displaced persons affected by humanitarian emergencies is often grim. Most have involuntarily fled their home from conflict, famine, political strife, natural disaster or a host of other reasons. Forced displacement is a broad term referring to individuals who have had to flee their homes involuntarily. The displacement can occur from naturally occurring events (e.g., an earthquake), man-made situations (e.g., re-located due construction of a dam), or from war or conflict. Internally displaced populations are those that have been forced to flee their homes but have remained within the boundaries of their own country; while refugees have fled across international boundaries due to persecution, war or violence [1,2]. This commentary primarily focuses on forced displacement due to conflict. The World Bank estimates that more than 1.5 billion people live in violent conflict-affected countries. Of the 35 countries listed by The World Bank as ‘fragile situations,’ 14 of the countries have conflict and are controlling malaria and one is a malaria-elimination country [3]. Populations affected by humanitarian emergencies face threats to security, an uncertain future and unreliable access to basic services including health care. When the emergency occurs in a malaria-endemic area, malaria-related deaths can and often do exceed those directly caused by the emergency [4]. Out of a total of 10,404,806 refugees protected and assisted by the United Nations High Commissioner for Refugees (UNHCR) globally in 2011, 7,019,383 (67.5%) live in areas in which malaria is either present throughout the year or occurs seasonally [5]. Historically, national malaria control strategic plans have not included displaced populations [6]. The focus on malaria elimination follows the same pattern, with little mention of how the goal of elimination would be effectively realized in areas hosting forcibly displaced populations. However, to control malaria in an equitable fashion and meet global targets, such as the Millennium Development Goals (MDGs), malaria elimination discussions need to systematically include persons affected and displaced by conflict. It is fitting to note that none of the low-income fragile or conflict-affected countries had achieved a single MDG as of April 2011 [3]. In the post-2015 UN Development Agenda debate currently on-going, conflict and fragility will be a component of 11 thematic consultations that are being organized by the United Nations Development Group to help address the failure of the MDGs in some of the lowest income countries and countries affected by conflict [7,8].

In October 2007, the Bill & Melinda Gates Foundation made an unexpected announcement regarding their goal of eradicating malaria [9]. This prompted lively debates in the malaria world regarding the feasibility of such a goal. What has been curiously lacking in these discussions has been the politically sensitive issue of how national and regional malaria elimination plans would include forcibly displaced persons who live in malaria endemic regions, including but not limited to refugees, internally displaced persons (IDPs), and asylum seekers. With rare exceptions [10-12], there has been little acknowledgement of forcibly displaced populations living in malaria-endemic countries or how to effectively ensure that these populations are included in the scaling up of interventions that are necessary to move toward malaria elimination.

Of the 100 malaria-endemic countries, 64 are controlling malaria and 36 are in some stage of elimination [13]. Of these, 30 malaria-controlling countries and 13 countries in some phase of elimination host displaced populations of ≥50,000 (see Additional file 1).

Of the 64 endemic countries that are controlling malaria, 56 (87.5%) host refugees, with 20 countries hosting between 20,000 to over 1,700,000 refugees. There are 35 (54.7%) malaria-controlling countries that host IDPs (including countries with IDPs that have undetermined population size, see Additional file 1), while 32 (50.0%) host both refugees and IDPs. Most of the 36 malaria-eliminating countries host displaced persons: 30 (83.3%) countries host refugees; 11 (30.6%) host IDPs; and 11 (30.6%) host both refugees and IDPs [5,13].

Considering the number of displaced persons in malaria-endemic countries, it becomes imperative to reframe the discussions about malaria elimination in such a way that these vulnerable populations are included. Discussions in global panels, reports and the published literature have focused on whether the goal of malaria elimination is achievable. Many malaria control experts have advocated for better and more sustainable health systems, improved malaria control programmes, and the need to recognize and include affected communities as partners in these efforts to meet this lofty goal [10,12,14-32]. While overall enthusiasm remains high for moving toward regional malaria elimination in areas that border high-transmission zones, cross-border collaboration will be difficult in situations of conflict, and persistent poverty, and scaling up of interventions will need to be modified for those contexts [10,11].

Multiple constraints currently affect the provision of malaria control, including but not limited to, a) costs of anti-malarial therapies; b) developing resistance to artemisinin (reflecting, in part, inappropriate use and monotherapy); c) inefficient drug delivery systems; d) widespread counterfeit drugs; e) poor infrastructure for health care facilities; f) weak health systems, especially with regard to surveillance and monitoring/evaluation; g) limited adequate diagnostics in many endemic areas; h) patient and provider beliefs that affect compliance with diagnostic test results, such as disbelieving negative malaria rapid diagnostic test (RDTs) results; i) lack of guidance for management of non-malarial fevers; j) limited arsenal of new anti-malarials; k) increased insecticide resistance; l) continued low levels of coverage of proven interventions, particularly in sub-Saharan Africa; m) lack of community engagement in malaria control activities; and, n) poor diagnosis and treatment in both public and private sectors [12,17,23,33-36]. These issues are common in many endemic areas and are heightened in countries hosting displaced persons, with the additional burden of the displaced individuals stressing fragile health care systems.

The challenges listed above are significant for national populations in endemic countries. They become daunting obstacles when endemic countries face complex humanitarian emergencies and the burden of hosting displaced persons. A sad reality is that politics often do not favour forcibly displaced populations, and countries can be loath to build or strengthen health care services in areas that host displaced persons for fear that it will send the message that the displaced are welcome to settle long term. Sociocultural and political questions abound regarding how to implement elimination in countries bordering “failed” states, countries in which portions of the territory are under guerrilla control or countries that are closed to outside intervention. It was noted in a panel at the 2010 meeting of the American Society for Tropical Medicine and Hygiene [37] that operational feasibility for elimination must include political, social and environmental factors, including the absence of conflict [38]. It is essential to have cross-border pacts and shared programmatic planning to have successful elimination programmes started and maintained [39].

It is necessary to understand the context of displaced persons to operationally plan for scale-up efforts that are needed for successful elimination programmes. They are often highly vulnerable, some without legal status, and many in war-torn regions. Often these are forgotten populations who languish in refugee camps for more than a decade (commonly referred to as ‘being warehoused’) and these protracted situations result in many displaced persons suffering from country-level lack of political will or attention to their plight [40]. The displaced are often located in marginal areas, where the surrounding host population itself is struggling to meet basic needs, and infrastructure and health care are absent or sorely in need. In addition to the traditional refugee camps, displaced persons also live in dispersed rural and urban settings, where significant barriers to access health services exist for a variety of reasons [41].

Funding for communicable disease control for displaced persons has historically not been a priority for many host countries and multilateral donors. Public health, moral and legal arguments can be and need to be made for the inclusion of displaced persons in national plans for malaria control, but this is rarely the case for both national strategic plans (NSPs) and proposals to The Global Fund to Fight AIDS, Tuberculosis and Malaria (Global Fund). A recent study showed that in African countries with refugee and/or IDP populations of ≥10,000 persons, refugees were included in malaria-related specific activities, programmes or funds in only 20% of NSPs and 11.3% of approved Global Fund proposals from Rounds 1–8 [6]. Out of the 30 countries with ≥10,000 refugees, only Sudan, Uganda and Kenya (10%) included refugee-specific activities in their NSPs. Malaria control activities that included IDPs were noted in 22.2% of NSPs and 12.5% of the approved Global Fund proposals [3]. Of the 21 countries with ≥10,000 IDPs, only Sudan and Uganda included IDP-specific activities (10%) in their NSPs. Scale-up strategies for displaced populations should be similar to the malaria control scale-up interventions targeted for the national population, although modified as necessary to take into account all the contextual factors seen in situations with forcibly displaced persons and surrounding host communities. These factors may include, for example, disruption to services secondary to insecurity, sudden movements of the displaced populations that make it difficult to ascertain the number of persons that need malaria control services, or sociocultural barriers that may impede treatment-seeking behaviour.

In the 2011, President’s Malaria Initiative country-level malaria operational plans, several countries described interventions, such as long-lasting, insecticidal nets (LLINs) or the use of indoor residual spraying, that were extended to refugee or IDP populations. Good examples of countries incorporating displaced populations include Senegal, where LLINs were distributed in conflict-affected areas and Ethiopia, where malaria RDTs were also procured for refugee-affected areas along the Sudanese and Somali borders.

In addition to the large amount of funding that is required for elimination efforts over time, the need for sustained political will at global, regional and country levels is a critical factor that will determine whether a country is willing to embark on and sustain the efforts needed for scaling up interventions to the level needed for elimination. Newman [42] stresses that if communities were empowered to be the focal centre of malaria control, political will would not fail. What was not defined in his argument was the term ‘community’ – does ‘community’ refer only to the national, mainstream population or does it actually encompass all elements of the community, including displaced populations that may or may not be nationals? Each country must do a thorough feasibility study that includes all facets of the population in order to determine if political, social, financial, operational and technical factors are sufficient to tackle elimination. On a pragmatic level, when examining the financial feasibility, it is important to acknowledge donor fatigue and the impact of a global economy that is weary from the financial strain of assisting so many persons affected by conflict. Furthermore, as funding to combat communicable diseases (e.g, HIV) reduces for a variety of reasons, including the current global financial difficulties, displaced persons may be disproportionately affected, as countries will prioritize their own citizens. As the world continues to struggle with increasing resource constraints, concerns have been voiced as to whether the reductions in malaria mortality and other public health gains are sustainable [7].

Malaria elimination is a worthy goal, and the challenges that lie ahead should not be underestimated. It is incumbent upon the global malaria community to expand the discourse on elimination to include the impact of displaced populations within malarious areas. The burden of these populations on the surrounding areas that host these populations has not been fully articulated. Spiegel *et al.* noted the difficulties in determining the magnitude of the problem in these populations, yet it is critical to strive to overcome these difficulties and estimate the size of the populations at risk for malaria in order to effectively plan the needed interventions [41]. This will include the need for better surveillance activities, including improving both data collection and analysis that is done in a timely manner to address fluid conflict situations. Sri Lanka can serve as a good case-study of a country that demonstrated positive malaria outcomes during a time of conflict by strengthening surveillance, case management and vector control activities utilizing resources from the government, external funding and partnerships with local Sri Lankan and international non-governmental organizations [43] (see Table 1).

**Table 1:** Summary and recommendations for policy makers

**Key points:**	
1	Forcibly displaced individuals, including refugees and internally displaced persons (IDPs), represent some of the poorest, most vulnerable populations that often lack a political and advocacy voice.
2	Of all refugees protected under the mandate of UNHCR, 67.5% are affected by malaria (approximately 7 million persons).
3	Countries affected by humanitarian emergencies and hosting displaced populations face significant obstacles in the implementation of effective malaria control and elimination strategies.
4	Rarely have displaced populations been included in national- and/or regional-level strategies.
5	Malaria control and elimination strategies require long-term political and financial commitment. Refugees, IDPs and other forcibly displaced persons run the risk of being further ignored in the current climate of decreasing global resources.
6	Equity in malaria control cannot be achieved without consideration of forcibly displaced populations.
**Key recommendations:**	
1	Acknowledge and address the impact of forcibly displaced populations on malaria control and elimination programmes.
2	Improve surveillance, data collection and analysis to better understand the magnitude of the problem (including national and forcibly displaced populations) in order to effectively plan and implement appropriate malaria control interventions.
3	Promote the active participation and partnership of the forcibly displaced population in malaria control programming and implementation.
4	Advocate for the forcibly displaced populations to improve equity when developing national- and regional-level strategies.

Without including the displaced persons in malaria control activities, the full impact of those interventions will not be realized. It is critical that displaced populations, whether they are refugees or IDPs, are considered as active partners in the delivery of interventions, not merely seen as beneficiaries. For example, a novel pilot programme for cross-border malaria control for IDPs was successfully organized and implemented by displaced persons in Myanmar, which demonstrated the feasibility of using a community-based organization to provide malaria control activities to a conflict-affected population [44,45].

Those affected by conflict and displacement often do not have a voice. They are some of the poorest people in the world, living in areas where the health care infrastructure and services are at a minimum. Conflict, famine and natural disasters will continue in the future, and complex issues such as global warming and shifting geopolitical realities may increase such situations. Now is the time for donors, ministries of health and, in particular, national malaria control programmes, and other implementing partners to start envisioning creative solutions that incorporate displaced populations into national and regional (i.e., cross-border) malaria control programmes. The post-2015 UN Development Agenda notes that there is tremendous work ahead for countries affected by conflict in order to make progress toward meeting the MDGs, as stagnation in this process has resulted, in part, from a failure to reach the most vulnerable populations [7]. Advocacy for these vulnerable but resilient populations must come from all involved stakeholders, including and perhaps most importantly, the affected populations, host governments and the donor community. Until equitable and sustainable malaria control includes everyone residing in endemic areas, the goal of malaria elimination will not be met.

## Competing interests

The authors declare that they have no competing interests.

## Authors’ contributions

HAW designed, drafted, and revised the manuscript. HH and PS drafted and revised the manuscript. All authors approved the manuscript.

## Supplementary Material

Additional file 1 Malaria controlling and malaria elimination countries* hosting displaced populations of ≥ 50,000. Click here for file
