# Supplementary material for: Discourse on malaria elimination: where do forcibly displaced persons fit in these discussions?
Source: Malar J. 2013 Apr 10;12:121. doi: 10.1186/1475-2875-12-121 (PMC3626721; doi:10.1186/1475-2875-12-121)
Supplement: Additional file 1 — Malaria controlling and malaria elimination countries* hosting displaced populations of ≥ 50,000. [file 1475-2875-12-121-S1.doc]

Table 1: Malaria Controlling and Malaria Elimination Countries* Hosting Displaced Populations of > 50,000

| **Countries Controlling Malaria** | **Total # Refugees and people living in refugee-like situations**  **UNHCR. Global Trends 2011. Table 1. Refugees, asylum-seekers, internally displaced persons (IDPs), returnees (refugees and IDPs), stateless persons, and other of concern to UNCHR by country/territory of asylum/end-2011 (pp. 38-41). http://www.unhcr.org/4fd6f87f9.html** (accessed 27 June 2012) | **Total # Internally Displaced Persons (IDPs)**  [**http://www.internal-displacement.org/8025708F004CE90B/%28httpPages%29/22FB1D4E2B196DAA802570BB005E787C?OpenDocument**](http://www.internal-displacement.org/8025708F004CE90B/(httpPages)/22FB1D4E2B196DAA802570BB005E787C?OpenDocument) (accessed 27 June 2012) | **Countries of Malaria Elimination** | **Total # Refugees and people living in refugee-like situa*t*ions**  **UNHCR. Global Trends 2011. Table 1. Refugees, asylum-seekers, internally displaced persons (IDPs), returnees (refugees and IDPs), stateless persons, and other of concern to UNCHR by country/territory of asylum/end-2011 (pp. 38-41).**  **http://www.unhcr.org/4fd6f87f9.html** (accessed 27 June 2012) | **Total # Internally Displaced Persons (IDPs)** [**http://www.internal-displacement.org/8025708F004CE90B/%28httpPages%29/22FB1D4E2B196DAA802570BB005E787C?OpenDocument**](http://www.internal-displacement.org/8025708F004CE90B/(httpPages)/22FB1D4E2B196DAA802570BB005E787C?OpenDocument) (accessed 27 June 2012) |
| --- | --- | --- | --- | --- | --- |
| **Afghanistan** | 3,009 | At least 448,000 | **Algeria** | 94,148 | Undetermined to 1,000,000 |
| **Bangladesh** | 229,669 | Undetermined | **Azerbaijan** | 1,730 | Up to 599,000 |
| **Burundi** | 35,659 | 78,800 | **China** | 301,018 |  |
| **Cameroon** | 100,373 |  | **Georgia** | 462 | Up to 258,000 |
| **Central African Republic (CAR)** | 16,730 | 105,000 | **Iran** | 886,468 |  |
| **Chad** | 366,494 | 126,000 | **Iraq** | 35,189 | 2,300,000 – 2,600,000 |
| **Colombia** | 219 | 3,876,000 – 5,454,766 | **Kyrgyzstan** | 6,095 | 67,000 |
| **Congo, Republic of** | 141,232 | Up to 7,800 | **Malaysia** | 86,680 |  |
| **Cote d’Ivoire** | 24,221 | At least 247,000 | **Mexico** | 1,677 | About 160,000 |
| **Democratic Republic of the Congo (DRC)** | 152,749 | About 710,000 | **South Africa** | 57,899 |  |
| **Ecuador** | 123,436 |  | **Sri Lanka** | 188 | About 125,000 |
| **Ethiopia** | 154,295 | Undetermined to 300,000 | **Thailand** | 89,253 | Undetermined |
| **India** | 185,118 | At least 506,000 | **Turkey** | 14,465 | 954,000 – 1,201,000 |
| **Indonesia** | 1,006 | Up to 180,000 |  |  |  |
| **Kenya** | 566,487 | About 250,000 |  |  |  |
| **Liberia** | 128,293 | Undetermined to 23,000 |  |  |  |
| **Mali** | 15,624 | 167,257 |  |  |  |
| **Countries Controlling Malaria** | **Total # Refugees and people living in refugee-like situations** | **Total # Internally Displaced Persons (IDPs)** | **Countries of Malaria Elimination** | **Total # Refugees and people living in refugee-like situations** | **Total # Internally Displaced Persons (IDPs)** |
| **Myanmar** |  | More than 458,000 |  |  |  |
| **Nepal** | 72,654 | About 50,000 |  |  |  |
| **Pakistan** | 1,702,700 | At least 900,000 |  |  |  |
| **Rwanda** | 55,325 | Undetermined |  |  |  |
| **Senegal** | 20,644 | 10,000 – 40,000 |  |  |  |
| **Somalia** | 2,099 | About 1,500,000 |  |  |  |
| **South Sudan** | 105,023 | Undetermined to 350,000 |  |  |  |
| **Sudan** | 139,415 | At least 2,200,000 |  |  |  |
| **Uganda** | 139,448 | About 30,000 |  |  |  |
| **United Republic of Tanzania** | 131,243 |  |  |  |  |
| **Venezuela** | 202,022 |  |  |  |  |
| **Yemen** | 214,740 | At least 463,500 |  |  |  |
| **Zimbabwe** | 4,561 | Undetermined to 570,000 |  |  |  |

*****Countries were defined as controlling or eliminating by the UCSF Global Health Group , Malaria Elimination Group (<http://malariaeliminationgroup.org/>), Atlas of Malaria Elimination Countries, 2011.
